# Relationship between regional relative theta power and amyloid deposition in mild cognitive impairment: an exploratory study

**DOI:** 10.3389/fnins.2025.1510878

**Published:** 2025-02-07

**Authors:** Jaesub Park, Woo Jung Kim, Han Wool Jung, Jae-Jin Kim, Jin Young Park

**Affiliations:** ^1^Department of Psychiatry, Yongin Severance Hospital, Yonsei University College of Medicine, Yongin, Gyeonggi, Republic of Korea; ^2^Institute of Behavioral Science in Medicine, Yonsei University College of Medicine, Seoul, Republic of Korea; ^3^Department of Psychiatry, Gangnam Severance Hospital, Yonsei University College of Medicine, Seoul, Republic of Korea; ^4^Center for Digital Health, Yongin Severance Hospital, Yonsei University Health System, Yongin, Gyeonggi, Republic of Korea

**Keywords:** source localization, amyloid PET imaging, EEG theta power, mild cognitive impairment (MCI), regional brain activity, Alzheimer’s disease biomarkers

## Abstract

**Introduction:**

Electroencephalographic (EEG) abnormalities, such as increased theta power, have been proposed as biomarkers for neurocognitive disorders. Advancements in amyloid positron emission tomography (PET) imaging have enhanced our understanding of the pathology of neurocognitive disorders, such as amyloid deposition. However, much remains unknown regarding the relationship between regional amyloid deposition and EEG abnormalities. This study aimed to explore the relationship between regional EEG abnormalities and amyloid deposition in patients with mild cognitive impairment (MCI).

**Methods:**

We recruited 24 older adults with MCI from a community center for dementia prevention, and 21 participants were included in the final analysis. EEG was recorded using a 64-channel system, and amyloid deposition was measured using amyloid PET imaging. Magnetic resonance imaging (MRI) data were used to create individualized brain models for EEG source localization. Correlations between relative theta power and standardized uptake value ratios (SUVRs) in 12 brain regions were analyzed using Spearman’s correlation coefficient.

**Results:**

Significant positive correlations between relative theta power and SUVR values were found in several brain regions in the individualized brain model during the resting eyes-closed condition, including right temporal lobe (*r* = 0.581, *p* = 0.006), left hippocampus (*r* = 0.438, *p* = 0.047), left parietal lobe (*r* = 0.471, *p* = 0.031), right parietal lobe (*r* = 0.509, *p* = 0.018), left occipital lobe (*r* = 0.597, *p* = 0.004), and right occipital lobe (*r* = 0.590, *p* = 0.005). During the visual working memory condition, significant correlations were found in both cingulate lobes (left: *r* = 0.483, *p* = 0.027; right: *r* = 0.449, *p* = 0.041), left parietal lobe (*r* = 0.530, *p* = 0.010), right parietal lobe (*r* = 0.606, *p* = 0.004), left occipital lobe (*r* = 0.648, *p* = 0.001), and right occipital lobe (*r* = 0.657, *p* = 0.001).

**Conclusion:**

The result suggests that regional increases in relative theta power are associated with regional amyloid deposition in patients with MCI. These findings highlight the potential of EEG in detecting amyloid deposition. Future large-scale studies are needed to validate these preliminary findings and explore their clinical applications.

## Highlights

• EEG theta power is considered a biomarker for cognitive impairment.
• There are regional correlations between theta activity and amyloid deposition.
• EEG has a potential to detect amyloid deposition.

## 1 Introduction

Electroencephalographic (EEG) abnormalities have been reported in various cognitive dysfunctions, such as dementia, mild cognitive impairment (MCI), and subjective cognitive decline (SCD), as well as neurocognitive disorders, including Alzheimer’s disease (AD) and Parkinson’s disease. These abnormalities, such as global slowing of EEG frequencies characterized by a relative increase in theta waves in the resting state, have been proposed as biomarkers for AD, the most prevalent form of dementia (Cassani et al., 2018). The slow-wave activity has also been proposed as a useful tool for the differential diagnosis of AD from other types of dementia (Livint Popa et al., 2021). Other EEG markers, such as low resting-state alpha power in patients with MCI, have been found to be more prominent in patients progressing to dementia, despite some inconsistencies (Lejko et al., 2020). Recently, with the widespread use of biomarkers related to amyloid pathology, EEG research has been conducted on patients with dementia or MCI diagnosed using biomarkers. One standard EEG study found a significant overall increase in theta band power at temporal electrodes for patients with AD and, to a lesser extent, those with MCI compared to healthy controls. Relative theta power was significantly correlated with several neuropsychological measures, but no significant correlation with cerebrospinal fluid (CSF) amyloid beta levels was observed (Musaeus et al., 2018). In a study of MCI and dementia where amyloid deposition was confirmed by CSF analysis or positron emission tomography (PET), a global slowing of oscillations (i.e., increased relative theta power) over time occurred in temporal and parieto-occipital electrodes as the disease progressed (Scheijbeler et al., 2023). Another study demonstrated that higher delta and theta powers were associated with clinical progression over time in a group of individuals with SCD and CSF amyloid pathology (Gouw et al., 2017). These theta wave abnormalities, which are thought to reflect underlying neurological dysfunction, have been reported to be associated with amyloid deposition in animal models. Animal EEG studies have demonstrated that the relative power of hippocampal theta has a positive correlation with amyloid-beta deposition in the hippocampus (Goutagny et al., 2013; Gutierrez-Lerma et al., 2013; Gauthier-Umana et al., 2020). The hippocampus is the main center of theta waves (Buzsáki, 2002; Karakas, 2020), and hippocampal hyperactivity is a major mechanism of brain toxicity in animal models of AD (Zott et al., 2019).

However, there is limited evidence regarding the relationship between EEG theta and amyloid deposition in human studies, making such a relationship remain controversial (Dubois et al., 2018; Teipel et al., 2018; Maestú et al., 2019; Yan et al., 2024). This discrepancy could be attributed to localized changes in the brain during the early stages of amyloid deposition, which are not sufficient to influence surface EEG recordings. In postmortem studies, initial amyloid deposition mainly starts at the basal portion of the brain and then spreads throughout the entire brain as the disease progresses (Thal et al., 2002); however, conventional EEG has poor spatial resolution, and measuring EEG waves arising from the base of the brain is challenging. Additionally, structural changes in dementia, such as increased CSF between the brain parenchyma and skull due to atrophy as the disease progresses, can skew EEG measurements. Such difficulties are also applicable to PET imaging. Despite recent advancements in amyloid PET, which enabled amyloid deposition to determine pathology, select high-risk groups, and measure treatment effectiveness (Vlassenko et al., 2012), identifying early localized amyloid deposition remains a challenge. The low spatial resolution of PET causes a partial volume effect (PVE), where the measured signal integrates contributions from both the target and surrounding tissues (Hoffman et al., 1979). This effect increases as the size of the region of interest becomes smaller. Consequently, identifying amyloid deposition status in early stage patients with only a small affected area becomes challenging. The low spatial resolution of EEG and amyloid PET makes studying the relationship between EEG abnormalities and amyloid deposition in humans difficult.

To address these challenges, studies using source localization techniques have been conducted to evaluate the relationship between regional abnormalities in brain activities and amyloid deposition. Magnetoencephalography (MEG) studies using various source localization techniques have shown that these limitations can be overcome by measuring electrical activity in deep brain structures, such as the hippocampus (Bénar et al., 2021). Additionally, various calculation methods to overcome PVEs in PET have been developed and applied, and recent studies have been actively conducted in patients in the early stages of amyloid deposition (Levin et al., 2021). Studies of neurocognitive disorders using this methodology have provided considerable information. In one MEG study, patients with amyloid-positive MCI had higher theta power in both hippocampi and higher classification accuracy than those with amyloid-negative SCD when using absolute theta band power in the right hippocampus (Luppi et al., 2022). Another study employing MEG and amyloid PET demonstrated that the effects of amyloid-beta deposition were reflected as increased alpha-band power in the medial prefrontal regions. Additionally, this study demonstrated that increased global theta power was associated with general cognitive decline and hippocampal atrophy, although not specific to amyloid-beta deposition (Nakamura et al., 2018). Despite the promising findings and advantages of MEG, large-scale studies are challenging owing to its low clinical and research accessibility.

Recently, with advances in computational modeling, efforts have been made to identify markers of amyloid deposition through EEG as well, measuring the local EEG of the brain by inversely calculating the EEG measured using surface electrodes and creating a three-dimensional (3D) model. In a study using exact low-resolution brain electromagnetic tomography (eLoreta) and CSF amyloid-beta levels, increased theta power in the forebrain regions was significant in patients with amyloid-positive MCI and suggested as an EEG marker for early AD (Cecchetti et al., 2021). Another study using 128-channel EEG and eLoreta reported a decrease in alpha and beta waves during the retention period of a working memory task in individuals with MCI. Additionally, this abnormality was correlated with a decrease in the size of the hippocampus, entorhinal cortex, and parahippocampal gyrus (Fodor et al., 2018). However, these studies have limitations due to the insufficient resolution for delicate source reconstruction or the absence of individual brain models constructed using magnetic resonance imaging (MRI) images or regional pathological information measured using amyloid PET. Therefore, additional studies are required to determine the relationship between regional EEG abnormalities and amyloid deposition in the human brain.

This study aimed to address these gaps by focusing on patients with MCI. We hypothesized that regional EEG abnormalities, such as increased theta power, are associated with regional amyloid deposition in patients with MCI. Patients with MCI are suitable for studying the relationship between regional EEG abnormalities and amyloid deposition because, although amyloid deposition has occurred, they have not yet developed significant brain atrophy that could affect EEG source localization. EEG signals in the resting state and during a visual working memory (VWM) task were measured and applied to an individualized brain model using the participant’s MRI to calculate the EEG source. The relative theta EEG power for each brain region was calculated for comparison with the local amyloid deposition measured using amyloid PET. Additionally, to further study the impact of the brain model using an individual’s brain structural image on EEG source localization, an additional analysis was conducted using only the general brain model.

## 2 Materials and methods

### 2.1 Participants

Between January 2017 and July 2017, 24 older adults with MCI were recruited through advertisements at a community center for dementia prevention and care services. All participants were registered in this public center following a neurocognitive function test and a psychiatrist’s clinical diagnosis. Mini-Mental State Examination (MMSE) (Kang et al., 1997) and Global Deterioration Scale (GDS) (Choi et al., 2002) scores were used by psychiatrists to confirm the clinical diagnosis of MCI. If the participant consented, we received the community center’s clinical report from a comprehensive neuropsychological battery, including the Korean Version of the Consortium to Establish a Registry for Alzheimer’s Disease Assessment Packet (Lee et al., 2002) and the Korean Version of Short Form Geriatric Depression Scale (SGDS-K) (Lee et al., 2013). The institutional review board of Gangnam Severance Hospital approved this study (2013-0104-008).

### 2.2 Methods

#### 2.2.1 Imaging data processing

##### 2.2.1.1 Image acquisition

**FIGURE 1:**
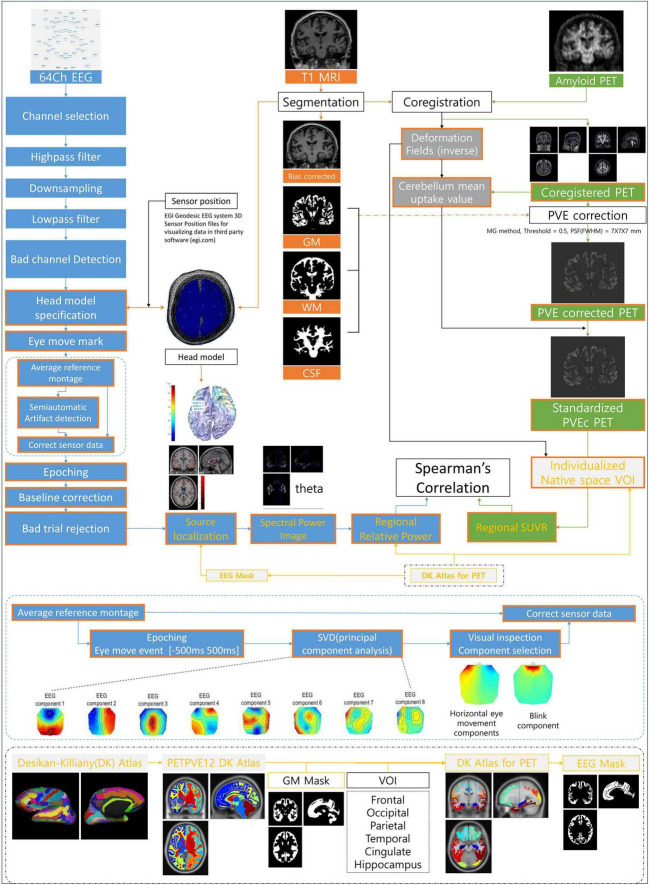
Illustration of the analysis process and method. EEG, electroencephalography; MRI, magnetic resonance imaging; PET, positron emission tomography; GM, gray matter; WM, white matter; CSF, cerebrospinal fluid; PVE, partial volume effect; PVEc, partial volume effect correction; VOI, volume of interest; SUVR, standardized uptake value ratio; DK Atlas, Desikan–Killiany Atlas; SVD, singular value decomposition.

Magnetic resonance imaging data were acquired using a 3T MRI scanner (Signa EXCITE, GE, Milwaukee, WI, USA). For sagittal 3D T1-weighted image (T1WI) with axial and coronal reconstruction, the imaging parameters were as follows: repetition time (TR) = 8.2 ms; echo time (TE) = 3.2 ms; slice thickness = 1.2 mm; flip angle = 12°; acquisition matrix = 256 × 256; and field of view (FOV) = 240 mm × 240 mm. All PET images were acquired using a Biograph mCT PET/CT scanner (Siemens Medical Solutions, Malvern, PA, USA). We intravenously administered 8.83 ± 1.56 mCi of 18F-florbetaben for amyloid PET. At 90 min after the injection of 18F-florbetaben, PET images were acquired for 20 min. After correcting for attenuation, scatter, and decay, 3D PET images were reconstructed using the ordered-subsets expectation maximization algorithm in a 256 × 256 × 223 matrix with a voxel size of 1.591 × 1.591 × 1 mm. Imaging data were processed using statistical parametric mapping (SPM12, Wellcome Trust Center for Neuroimaging, London, UK) and the PETPVE12 toolbox^1^ in MATLAB R2021 (MathWorks, Natick, MA, USA). The preprocessing and multimodal analysis process of EEG, PET, and MRI data is as follows, and the flow chart is shown in Figure 1.

##### 2.2.1.2 Magnetic resonance imaging processing

Magnetic resonance imaging scans were automatically segmented into gray matter (GM), white matter (WM), and CSF partitions using the tissue probability maps from SPM12. The partitions of each participant in native space were subsequently registered to the routine MNI template of SPM12. In this process, two files containing individual reverse and forward normalization parameters (deformity fields) were generated for imaging analysis.

##### 2.2.1.3 PET data processing

Each participant’s amyloid PET scans were co-registered to a bias-corrected image of the corresponding structured MRI scan using SPM12 function and visual inspection. Correction for the PVE was performed using the algorithm proposed by the Müller–Gartner method (PVEc-MG method) (Muller-Gartner et al., 1992), which was implemented using the PETPVE12 toolbox. The PVEc-MG method is a three-compartment PVEc method that discriminates signals from the brain GM, WM, and CSF. It is one of the most widely used MRI-based methods for PET image analysis (Erlandsson et al., 2012). Briefly, this method assumes that the observed PET signal from a GM voxel is a spatially weighted average of the actual tracer uptake signal at the GM voxel and signals from the surrounding WM and CSF. Spatial weights were determined using the point-spread function of the PET scanner. The proposed PVEc algorithm corrected for the spill-out effect of signal leakage from the GM to the surrounding tissue as well as the spill-in effect of signal from the surrounding tissue into the GM compartment. Tracer activities in the WM and CSF were assumed to be homogeneous in each compartment.

##### 2.2.1.4 Extraction of regional imaging features

Regional amyloid PET uptake values were sampled from 12 brain regions defined in the Desikan–Killiany Atlas (Desikan et al., 2006) (an atlas included in the PETPVE12 toolbox; the original brain atlas was propagated to the MNI space). The Desikan–Killiany Atlas, which covers the entire cerebral cortex as well as the hippocampus, is widely used for amyloid PET studies including amyloid PET staging (Mattsson et al., 2019). We only used the cerebral lobes and hippocampus despite the atlas providing volumes of interest (VOIs) smaller than the cerebral lobe and subcortical regions. This choice was made because 3D reconstructed EEG images have relatively low spatial resolution and do not include subcortical regions, except for the hippocampus. The atlas labels were multiplied by the reference template’s binary GM mask with a threshold of a 50% GM probability. The atlas in the reference space was transformed into each participant’s native space using inverse deformity fields. The regional mean standardized uptake ratios of participants were obtained using the function implemented in the PETPVE12 toolbox. Similar to previous studies, the mean uptake value of the entire cerebellum was extracted from the PVE-uncorrected PET image and used as the reference region (Teipel et al., 2015; Grothe et al., 2017; Levin et al., 2021; Ota et al., 2022). The uptake value of voxels was subsequently converted to standard uptake value ratios by scaling to the mean uptake of the whole cerebellum in the non-PVE-corrected data.

#### 2.2.2 Electroencephalogram processing

##### 2.2.2.1 Electroencephalogram recording

Electroencephalographic was recorded using a 64-channel electrode system (Geodesic Sensor Net, Electrical Geodesics, Inc., Eugene, OR, USA), referenced to the vertex electrode (Cz). Before starting the EEG recording, participants were instructed to stay awake during the recording period. EEG was measured for >5 min for each condition while monitoring whether the participants were awake. Four electrodes (E61, E62, E65, and E64) were used as electrooculogram (EOG) electrodes for vertical and horizontal eye movement detection.

##### 2.2.2.2 Recording procedure

**FIGURE 2:**
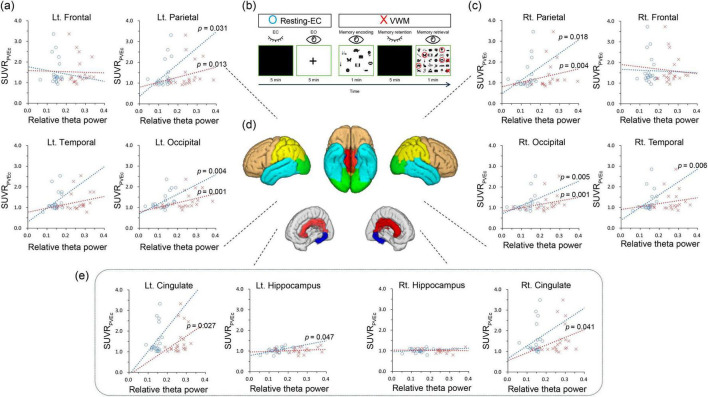
Correlations between the standardized uptake value ratio and relative theta power in resting EC and VWM states (individualized brain model). **(B)** The session consists of resting-EC (eye-closed and eye-open) and VWM (memory encoding, memory retention, and memory retrieval) tasks. **(A,C,E)** Blue circles indicate resting EC condition, while red Xs denote VWM condition. Dotted lines represent trend lines. **(D)** The brown color represents the frontal lobe, turquoise indicates the temporal lobe, yellow the parietal lobe, and green the occipital lobe. Red corresponds to the cingulate lobe, and blue to the hippocampus. Resting-EC, resting-state eye closed condition; VWM, visual working memory condition; SUVR_PVEc_: partial volume effect corrected standardized uptake value ratio.

The EEG recording procedure consisted of a single session lasting approximately 15 min. The session commenced with a 5-min resting-state EEG recording, during which the participants were seated in a chair with their eyes closed. Clear instructions were given to ensure wakefulness during this period. After 1 min of rest, EEG data were recorded for 7 min while participants performed a VWM task. During this task, participants were presented with a piece of paper containing 10 target items, which they were instructed to encode within 1 min. Following the encoding phase, participants closed their eyes and retained the 10 items in their minds for 5 min. The final segment of the session was allotted 1 min for participants to recall and mark the 10 target items out of 30 problem items (Figure 2B). In this study, brain waves during the resting (resting-EC condition) and retention periods (VWM condition) were analyzed. This has been explained in detail in another study (Ahn et al., 2022).

##### 2.2.2.3 Electroencephalogram preparation

Raw EEG data were high-pass filtered using a fifth-order Butterworth filter at 1 Hz, down-sampled to 200 Hz, and low-pass filtered using a fifth-order Butterworth filter at 60 Hz. The beginning and end of the EEG data were cropped for 10 s. A channel was deemed bad if over 20% of the data had a *z* score > 5 SD for 100 ms or >80 μV for 1,000 ms. In the case of a bad channel, the problem of data recording was confirmed through visual inspection. If no issues were observed with the data file itself, the following analysis was performed.

##### 2.2.2.4 Head model specification

For preprocessing suitable for dipole source reconstruction analysis, head model specification was performed using two methods included in SPM12. First, in the personalized head model, each participant’s T1 structural MRI images were segmented to create an individual brain mesh consisting of the skin, skull, CSF, and brain. Alternatively, employing the generalized head model, an existing brain mesh based on the standard MNI brain served as the foundation in SPM. Following the creation of these head mesh models, all subsequent preprocessing and analysis steps were meticulously replicated for both the personalized and generalized head models. Using the methods in SPM12, three fiducials (the nasion and the left and right pre-auricular points) were marked on each mesh. Using the average 3D coordinate data of 64 electrodes and the fiducials of the HydroCel Geodesic Sensor Net from the EGI, the electrode positions were co-registered on individual meshes.

##### 2.2.2.5 Semiautomatic eye movement artifact correction and automatic artifact rejection

Using the eyeblink function included in SPM12, we set the threshold to four and marked the artifact events in the EOG channel data. EEG channel data were re-referenced using the average reference montage. In this re-referenced EEG data, 500 ms before and after the eye-moment event was epoched. For these epochs, the components were separated using principal component analysis, and eye blink or horizontal eye movement components were selected through visual inspection. The previously re-referenced EEG data were corrected using the selected eye-movement component data. The eye movement-corrected EEG was epoched into 2,000-ms trials, and baseline correction was performed using the first 100 ms as the baseline. Trials were rejected if >20% of the total had a *z* score of >5 SD for 100 ms or >80 μV for 1,000 ms.

##### 2.2.2.6 Three-dimensional reconstruction and power spectrum analysis

Electroencephalogram 3D reconstruction was performed using head models and artifact-free EEG data. During this process, an EEG mask corresponding to GM was created using an atlas designed for PET analysis. Spectral power analysis was subsequently performed (delta, 1–4 Hz; theta, 4–8 Hz; alpha, 8–13 Hz; and beta, 13–30 Hz). Because the analyzed data file was an image file of the same form as MRI or PET, the subsequent analysis underwent the same process as ordinary image analysis. The same atlas and method used for PET analysis were applied to obtain the mean power value for each brain region, and the subsequent relative theta power values were calculated for each area. Relative theta power was obtained by dividing the mean value of the theta power image by the sum of the mean values of each power image.

### 2.3 Statistical analysis

Statistical analyses were performed using IBM SPSS software, version 23 (IBM Corp., Armonk, NY, USA). Statistical significance was set at *p* < 0.05. Correlations between SUVR, relative theta power values and MMSE were analyzed in 12 regions of interest from the left and right among the 6 regions, including the hippocampus and 5 cortical lobes (Figure 2D). Considering the relatively small number of participants, Spearman’s correlation coefficient was used. No multiple-comparison corrections were performed for exploratory purposes.

## 3 Results

A total of 24 participants were enrolled in this study; however, three individuals withdrew their participation during the study owing to the inconvenience of multi-imaging. Thus, 21 participants who completed all the assessments were analyzed. All 21 patients were diagnosed with mild cognitive decline by psychiatrists in community healthcare centers. Of the 19 patients who agreed to provide neuropsychological test results, one was illiterate and three met the cut-off score of the SGDS-K. The mean age was 76.3 (±3.6) years, and the mean MMSE and GDS scores were 24.2 (±2.7) and 3.0 (±0.5), respectively. The clinical reading of the amyloid PET results by a radiologist was negative, positive, and unclear in 14, 6, and 1 participant(s), respectively (Table 1).

**TABLE 1:** Clinical characteristics of the participants.

	Total (*N* = 21)
Age (years)	76.3 (±3.6)
Education years^1^	8.1 (±4.8)
**Sex**	
Female	13 (61.9%)
Male	8 (38.1%)
MMSE	24.2 (±2.7)
GDS	3.0 (±0.5)
**Handedness**	
Right	20
Left	1
**Amyloid PET^2^**	
Positive	6
Negative	14
Uncertain	1

MMSE, Mini-Mental State Examination; GDS, Global Deterioration Scale; PET, positron emission tomography.

^1^One participant refused to disclose their education level.

^2^Classification based on the clinical assessment by the radiologist.

According to the correlation analysis, in the resting eyes-closed (EC) condition of the individualized brain model, a significant positive correlation was observed between relative theta power and SUVR values in the right temporal lobe (*r* = 0.581, *p* = 0.006), the left hippocampus (*r* = 0.438, *p* = 0.047), both parietal lobes (left: *r* = 0.471, *p* = 0.031; right: *r* = 0.509, *p* = 0.018), and both occipital lobes (left: *r* = 0.597, *p* = 0.004; right: *r* = 0.590, *p* = 0.005). In the VWM condition of the individualized brain model, a significant positive correlation was found between relative theta power and SUVR values in both cingulate lobes (left: *r* = 0.483, *p* = 0.027; right: *r* = 0.449, *p* = 0.041), both parietal lobes (left: *r* = 0.530, *p* = 0.01; right: *r* = 0.606, *p* = 0.004), and both occipital lobes (left: *r* = 0.648, *p* = 0.001; right: *r* = 0.657, *p* = 0.001) (Table 2 and Figure 2). In the resting EC condition of the general brain model, a significant positive correlation was observed between relative theta power and SUVR values in the right parietal lobe (*r* = 0.448, *p* = 0.042), and both occipital lobes (left: *r* = 0.532, *p* = 0.013; right: *r* = 0.606, *p* = 0.004). In the VWM condition of the general brain model, a significant positive correlation was found between relative theta power and SUVR values in both temporal lobes (left: *r* = 0.544, *p* = 0.011; right: *r* = 0.499, *p* = 0.021), both parietal lobes (left: *r* = 0.453, *p* = 0.039; right: *r* = 0.491, *p* = 0.024), and both occipital lobes (left: *r* = 0.675, *p* = 0.001; right: *r* = 0.639, *p* = 0.002) (Table 2). In correlation analyses with MMSE, neither SUVR values nor relative theta power in any brain region were statistically significant.

**TABLE 2:** Correlation analysis between regional relative theta power and SUVR from amyloid PET in different conditions and brain models.

		Individualized brain model				General brain model			
		Resting-EC		VWM		Resting-EC		VWM	
Regions		*r*	*p*-Value	*r*	*p*-Value	*r*	*p*-Value	*r*	*p*-Value
Frontal lobe	Lt	−0.105	0.650	0.258	0.258	−0.194	0.401	0.308	0.175
	Rt	−0.008	0.973	0.184	0.424	−0.045	0.845	0.243	0.289
Cingulate lobe	Lt	0.282	0.216	0.483*	0.027	0.204	0.375	0.392	0.079
	Rt	0.095	0.683	0.449*	0.041	0.106	0.646	0.369	0.100
Temporal lobe	Lt	0.295	0.195	0.374	0.095	0.266	0.243	0.544*	0.011
	Rt	0.581**	0.006	0.300	0.186	0.427	0.053	0.499*	0.021
Hippocampus	Lt	0.438*	0.047	0.162	0.482	0.413	0.063	0.287	0.207
	Rt	0.019	0.933	−0.047	0.841	0.060	0.797	0.162	0.482
Parietal lobe	Lt	0.471*	0.031	0.530*	0.013	0.408	0.067	0.453*	0.039
	Rt	0.509*	0.018	0.606**	0.004	0.448*	0.042	0.491*	0.024
Occipital lobe	Lt	0.597**	0.004	0.648**	0.001	0.532*	0.013	0.675**	0.001
	Rt	0.590**	0.005	0.657**	0.001	0.606**	0.004	0.639**	0.002

SUVR, standardized uptake value ratio; PET, positron emission tomography; Lt, left; Rt, right; Resting-EC, resting-state eye closed condition; VWM, visual working memory condition.

*Significant at *p* = 0.05.

**Significant after Bonferroni correction (*p* = 0.05/6).

## 4 Discussion

In this study, the hypothesis was substantiated by the observed correlation between theta power and amyloid deposition in various brain regions. Notably, the manifestation of these relationships differed in each region and varied depending on the type of task and brain model employed.

The increase in theta power, which coincided with regional amyloid deposition observed in this study, is consistent with previous findings. The hippocampus, as the center of theta wave oscillations, shows increased theta wave activity due to amyloid deposition. Therefore, region-related theta wave abnormalities in the cortex may be due to functional changes within the hippocampus itself, affecting neural activity in other brain regions interconnected with the hippocampus. In contrast, in human studies, interactions within neocortical laminar structures are known to modulate theta waves, as evidenced by stereotactic EEG research on patients with epilepsy. This previous research showed that laminar structures in the inferotemporal, perirhinal, entorhinal, prefrontal, and anterior cingulate cortices are capable of generating both spontaneous and cognitive task-evoked local theta potentials (Halgren et al., 2015). Animal studies suggest that neocortical amyloid deposition, occurring before neural death, disrupts these laminar interactions through synaptic disconnection, impacting the electrical activity of pyramidal cells (Lison et al., 2014). Similarly, it can be hypothesized that regional amyloid deposition in the human neocortex may also lead to regional anomalies in theta wave activity.

These areas, encompassing the hippocampus, cingulate, and parahippocampal regions within the temporal lobe, are postulated to constitute brain circuits integral to memory, specifically the Papez circuit, and are known to have structural abnormalities in MCI (Forno et al., 2021). Especially, the correlations between EEG abnormalities and amyloid deposition were prominent in the cingulate during the VWM state. Such a result is expected to derive from the role of anterior cingulate in memory or attention tasks (Davis et al., 2000; Luerding et al., 2008). In a study involving healthy adults, researchers used a visuospatial memory task to calculate EEG sources through a 256-channel high-density EEG and a general brain model. They found that theta wave activity in the cingulate increased when the quality of memory representations was lower or less accurate (Muthukrishnan et al., 2020). In the present study, this increase in cingulate theta waves during memory tasks in patients with MCI reflects a decline in the quality of memory representations, suggesting a possible association with regional amyloid deposition. Considering that anterior cingulate cortex cooperates with prefrontal cortex or subcortical areas as shown in functional connectivity during memory or attention-shifting tasks (Kondo et al., 2004; Lenartowicz and McIntosh, 2005), the EEG abnormalities in the VWM task state may indicate cognitive impairment that is more prominently shown in cognitive burden. Also, in this study, the correlation between brain activity and amyloid deposition varied according to the task performed in the temporal lobe and the hippocampus. This variability may be due to the brain compensating for amyloid-related abnormalities during task performance, particularly with a lower amyloid burden. A study on SCD reported a nonlinear relationship between amyloid burden and EEG abnormalities, which followed a U-shaped curve for delta power or an inverted U-shaped curve for other powers. It was suggested that an early compensatory mechanism could exist based on the relatively low amyloid burden (Gaubert et al., 2019). Although this study did not explore these mechanisms in depth, it highlights the need for future research to investigate how specific tasks can reveal changes in brain activity related to amyloid deposition.

The frontal lobe showed no correlation in any condition or analysis method despite amyloid deposition. This is consistent with previous studies reporting increased resting theta power in the parietal, occipital, and right parietal regions, excluding the frontal lobe, in patients with AD compared to individuals without AD (Olgun et al., 2024). On the other hand, this lack of correlation may be due to the possibility that the tasks or conditions used are not related to frontal lobe function. Studies using rats have shown that amyloid deposition in the hippocampus is manifested by changes in alpha or beta waves in the frontal lobe (Hidisoglu and Chiantia, 2023). However, in the present study, no correlation was observed between alpha and beta waves in the frontal lobe. Additional studies using various tasks are needed to identify EEG markers that correlate with amyloid deposition in the frontal lobe.

This study also suggested that incorporating an individualized brain model can improve the prediction of regional amyloid deposition using EEG, as the approach yielded more significant results in various regions, including the cingulate cortex, temporal lobe, hippocampus, and parietal lobe. When employing a brain model based on a generic average brain structure, the occipital and parietal lobes exhibited correlations similar to those in the model reflecting individual brain structures. Conversely, regions such as the temporal, cingulate, and hippocampus, which correspond to the basal and medial portions of the brain, displayed disparate results. This could be due to these areas being pathways for signals traversing a substantial portion of the brain, making them more susceptible to individual structural variations (Krishnaswamy et al., 2017). This insight suggests that research approaches might need to be tailored according to the study subject. For instance, studies focusing on early AD or early-stage conditions such as SCD may require models that incorporate individual brain structures. Conversely, for patients with MCI or late-stage AD with advanced amyloid deposition, a standard model may be more suitable for broad-scale research.

In fact, whether brain waves generated in subcortical structures, such as the hippocampus, can be accurately calculated using EEG or MEG, which are measured from the surface, remains a very controversial issue. In studies targeting patients where actual brain waves were accurately measured by inserting electrodes into the hippocampus or its surroundings for deep brain stimulation, some reports showed that MEG or EEG was measured with high accuracy (Pizzo et al., 2019; Seeber et al., 2019), while others found them to be very inaccurate (Fahimi Hnazaee et al., 2020). Some reports suggest that the accuracy of EEG and MEG differs depending on the direction and depth of the dipole (Piastra et al., 2021). Additionally, in the present study, the average relative power value of the entire hippocampus region was obtained using smoothed images rather than single dipoles. Therefore, additional research is needed to determine whether this approach accurately reflects actual hippocampal electrical activity.

Despite its advantages, this study has several limitations. First, the study involved patients diagnosed with MCI based on clinical symptoms in a community center, including many participants who showed subthreshold amyloid deposition with negative amyloid PET findings. Therefore, the observed relationship between amyloid deposition and EEG abnormalities cannot be generalized to AD. Second, although this study used a 64-channel EEG and average sensor location data similar to actual medical examinations, >128 channels and individual sensor location measurements are warranted to calculate the EEG source accurately (Liu et al., 2017). This study has the advantage of confirming meaningful results using equipment applicable in the medical field. However, for actual applications, studies comparing differences between multi-channel and individual sensor position measurements are warranted. Third, although an automatic artifact removal method was used for EEG analysis, visual inspection was used for the principal component analysis. This may limit objectivity and reproducibility owing to the experience and judgment of the analyst in terms of clinical application. Fourth, the issue of multiple comparisons is yet to be overcome. In some ROIs, significant results were observed even when Bonferroni correction was applied (*p* < 0.008); however, when task and brain model conditions were considered, achieving statistical significance remained challenging. Although a very large-scale study is necessary to confirm statistical significance, this study serves as an exploratory investigation that lays the foundation for future research. Finally, correlations with detailed neuropsychological tests including detailed depression symptom assessment were not analyzed. It may have been difficult to measure the subtle cognitive decline of MCI for the purpose of MMSE to screen for general cognitive decline. In addition, although MCI was diagnosed as clinically separate from depression, there is a limitation that the influence of depression cannot be completely ruled out.

This study found that regional theta waves measured using the source localization method were associated with increased regional amyloid deposition. In addition, these association patterns may vary depending on the cognitive task performed. Although this is an exploratory study that needs to be confirmed through studies with higher-resolution EEG devices, larger sample size, more robust prediction models, and broader cognitive function groups including SCD and dementia, the current results show some potential of EEG in measuring amyloid deposition without the radiological concerns of amyloid PET that may help in repeated monitoring during long-term treatment or in screening large populations for treatment. We anticipate that this will be particularly advantageous in contemporary practices, especially with the recent introduction of a fundamental treatment method for amyloid plaque removal.

## Data Availability

The raw data supporting the conclusions of this article will be made available by the authors, without undue reservation.

## Footnotes

1
https://github.com/GGonEsc/petpve12
